# Prediction of lymphovascular space invasion using a combination of tenascin-C, cox-2, and PET/CT radiomics in patients with early-stage cervical squamous cell carcinoma

**DOI:** 10.1186/s12885-021-08596-9

**Published:** 2021-07-28

**Authors:** Xiaoran Li, Chen Xu, Yang Yu, Yan Guo, Hongzan Sun

**Affiliations:** 1 Department of Radiology, Shengjing Hospital of China Medical University, Shenyang, Liaoning China; 2 GE Healthcare, Shenyang, Liaoning China

**Keywords:** PET/CT, Radiomics, Machine learning, Lymphovascular space invasion, Cervical squamous cell carcinoma

## Abstract

**Background:**

Lymphovascular space invasion is an independent prognostic factor in early-stage cervical cancer. However, there is a lack of non-invasive methods to detect lymphovascular space invasion. Some researchers found that Tenascin-C and Cyclooxygenase-2 was correlated with lymphovascular space invasion. Radiomics has been studied as an emerging tool for distinguishing tumor pathology stage, evaluating treatment response, and predicting prognosis. This study aimed to establish a machine learning model that combines radiomics based on PET imaging with tenascin-C (TNC) and cyclooxygenase-2 (COX-2) for predicting lymphovascular space invasion (LVSI) in patients with early-stage cervical cancer.

**Methods:**

One hundred and twelve patients with early-stage cervical squamous cell carcinoma who underwent PET/CT examination were retrospectively analyzed. Four hundred one radiomics features based on PET/CT images were extracted and integrated into radiomics score (Rad-score). Immunohistochemical analysis was performed to evaluate TNC and COX-2 expression. Mann-Whitney U test was used to distinguish differences in the Rad-score, TNC, and COX-2 between LVSI and non-LVSI groups. The correlations of characteristics were tested by Spearman analysis. Machine learning models including radiomics model, protein model and combined model were established by logistic regression algorithm and evaluated by ROC curve. Pairwise comparisons of ROC curves were tested by DeLong test.

**Results:**

The Rad-score of patients with LVSI was significantly higher than those without. A significant correlation was shown between LVSI and Rad-score (*r* = 0.631, *p* < 0.001). TNC was correlated to both the Rad-score (*r* = 0.244, *p* = 0.024) and COX-2 (*r* = 0.227, *p* = 0.036). The radiomics model had the best predictive performance among all models in training and external dataset (AUCs: 0.914, 0.806, respectively, *p* < 0.001). However, in testing dataset, the combined model had better efficiency for predicting LVSI than other models (AUCs: 0.801 vs. 0.756 and 0.801 vs. 0.631, respectively).

**Conclusion:**

The machine learning model of the combination of PET radiomics with COX-2 and TNC provides a new tool for detecting LVSI in patients with early-stage cervical cancer. In the future, multicentric studies on larger sample of patients will be used to test the model.

**Trial registration:**

This is a retrospective study and there is no experimental intervention on human participants. The Ethics Committee has confirmed that retrospectively registered is not required.

## Background

According to current global cancer statistics, cervical cancer ranks second among female tumors, and the number of new cases in developing countries is increasing annually and involving younger women [1]. Some studies have shown that LVSI is an independent prognostic factor in early-stage cervical cancer [2–6]. LVSI was detected in 17.8% of early-stage cervical cancer cases in the initial pathological examination [7]. Herr et al. [3] found that the presence of satellite LVSI was associated with significantly reduced overall survival and disease-free survival, compared with lack of any LVSI. According to Memarzadeh et al. [4], 86% of patients with perineural involvement in the parametria had evidence of parametrial LVSI, and the multivariate analysis revealed that large tumor size (> 4 cm), parametrial perineural invasion, cervical LVSI, and tumor depth (> 2/3) were significant simultaneous predictors of recurrence for early-stage cervical cancer (*p* < 0.05). Subsequently, Pol et al. [5] also confirmed that conjoined and satellite LVSIs were significantly associated with recurrence and survival. In a retrospective cohort study, LVSI was shown to be an independent factor that affects overall survival (*p* = 0.009) and progression-free survival (*p* = 0.006) in patients with early-stage cervical cancer [6]. However, LVSI could only be confirmed by postoperative pathology.

In recent years, radiomics based on PET/CT imaging has been studied as an emerging tool for distinguishing tumor pathology stage, evaluating treatment response, and predicting prognosis [8, 9]. Lambin et al. [10] proposed the radiomics hypothesis that intratumoral heterogeneity evaluated by imaging could be the expression of genomic heterogeneity, as tumors with genomic heterogeneity are more likely to metastasize.

In previous studies, some protein molecules expression have been shown to be associated with lymphangiogenesis, lymph node metastasis [11], and lymphovascular invasion in the early-stage cervical cancer. Hoellen et al. [12] determined that cyclooxygenase-2 (COX-2) expression was significantly associated with LVSI (*p* = 0.017). The expression of tenascin-C (TNC) in invasive cervical carcinoma was markedly increased [13]. Pilch [13] et al’s research showed that, in 84% of the cases examined, a strong TNC immunoreactivity was noted around and within the tumor cell nests. TNC is not only associated with epithelial–mesenchymal transition, proliferation, and migration of cancer cells, but it also facilitates the formation of cancer stroma, including desmoplasia and angiogenesis [14]. Thus, we aimed to explore the association of radiomics derived from ^18^F-fluorodeoxyglucose PET/CT imaging combined with COX-2 and TNC expressions with LVSI in early cervical cancer and to establish a machine learning model of the combination of PET/CT radiomics, COX-2, and TNC to predict LVSI in patients with early-stage cancer.

## Patients and methods

### Patient cohort

This monocentric and retrospective study was performed at the Department of Radiology at Shengjing Hospital in Shenyang, China. Between January 2015 and December 2019, 131 female patients with cervical cancer confirmed by biopsy pathology underwent pretreatment ^18^F-FDG PET/CT. Inclusion criteria: (1) histologically confirmed cervical cancer with stage Ia–IIa determined by the 2018 International Federation of Gynecology and Obstetrics (FIGO) classifications [15]; (2) absence of other malignant tumors; (3) normal serum glucose levels before undergoing PET/CT. Of these patients, 19 subjects were excluded because of previous chemoradiotherapy before the examination (*n* = 5), tumor volume < 1 cm^3^ leading to image data being unsuitable for textural feature measurement (*n* = 10), and surgery performed in another hospital (*n* = 4). Finally, 86 patients (42 LVSI and 44 non-LVSI) were randomly divided into two groups, including the training and testing datasets, according to a 7:3 ratio. An additional 26 patients were used as an external dataset for model validation.

### Immunohistochemistry

All tissue specimens of cervical carcinoma were prepared by the Department of Pathology in our hospital. Immunohistochemical staining was performed using Leica BOND MAX™ (Leica Biosystems, Shanghai, China). For immunohistochemical detection of COX-2 and TNC protein expressions, the sections were incubated with goat anti-human COX-2 (1:400 dilution) or rabbit anti-human TNC (1:400 dilution) polyclonal primary antibodies (both from Abcam, Shanghai, China) at 4 °C overnight. After washing, the sections were incubated with species-appropriate enzyme-conjugated anti-rabbit and anti-goat secondary antibodies. Dewaxing, antigen epitope exposure, blocking, incubation with primary antibody, development of diaminobenzidine oxidation color, hematoxylin staining, and dehydration were automatically completed by the computer.

After the tissue was sliced, it was placed on Pannoramic MIDI tissue slice scanner (3DHISTECH Ltd., Budapest, Hungary), which simultaneously moved and scanned the image, forming a file that contained all the information on the tissue section. The file could be magnified 1–400 times using the Pannoramic viewer software, and the picture could be intercepted at any location. The QuantCenter (3DHISTECH Ltd.) is an analysis software that supports the Pannoramic viewer. After image scanning is completed, the DensitoQuant software in the QuantCenter automatically recognizes and sets all dark brown areas on the tissue section as strong positive, brown yellow as moderate positive, light yellow as weak positive, and blue cell nucleus as negative. Furthermore, all strong-positive, moderate-positive, weak-positive, and negative areas (in pixels); the percentage of positive areas; and the H-score (Immunohistochemical score) were analyzed for each tissue [16]. According to the previous study, H-score = staining intensity [negative (0), mild (+ 1), moderate (+ 2), intense (+ 3)] * percentage (%) of positive stained cells [negative (0), < 5% positive stained cells (+ 1), 5–20% cells positivity (+ 2), 21–50% cells positivity (+ 3), (> 50% cells positivity (+ 4)]. All cases were divided into three groups according to the extent of staining and H-score: negative or weak-positive(H-score, 1 and 2), moderate-positive(H- score,3,4 and 6), and strong-positive groups(H-score,8,9 and 12) [17].

### ^18^F-FDG PET/CT acquisition and features extraction

The patients rested quietly for 60 min before PET/CT (Discovery PET/CT 690; GE Healthcare, Chicago, IL, USA) scanning. All CT and PET scans were acquired with free breathing for attenuation correction and image fusion. First, low-dose non-enhanced CT images were acquired with a bulb voltage of 120 kV, auto mA (30–210 mA; noise index, 25), and slice thickness of 3.27 mm. Then, the PET data were acquired after CT scanning using a three-dimensional acquisition mode at a speed of 1.5 min/bed (7–8 beds in total) and a matrix size of 192 × 192. The time-of-flight and point-spread function techniques were also used in the reconstruction. A volume of interest (VOI) of tumor was automatically obtained on an AW4.5 workstation (GE Healthcare) using a threshold of 42%SUVmax. The metabolic tumor volume (MTV), total lesion glycolysis (TLG), and maximum (SUV_max_), mean (SUV_mean_), and peak (SUV_peak_) standard uptake values were measured automatically corrected with body weight inside the segmented VOI.

The VOI based on PET images was manually drawn layer by layer around the cervical squamous cell carcinomas by two nuclear medicine physicians, who performed the task independently. Both had more than 10 years of experience and were blinded to patients’ clinical data. In order to ensure the stability of segmentation, every nuclear medicine physician independently segmented VOI of all images. Then, 40 VOIs of PET image from two doctors were randomly selected to extract radiomics features and calculate intraclass correlation coefficient (ICC). In the end, the VOIs of one doctor were randomly selected to be used for research. The radiomics features were extracted from the PET images using AK software (Artificial Intelligence Kit, GE Healthcare, Shanghai, China) [18].

### Calculation of Radiomics score

All data analyses were processed with R version 3.5.1 (The R Foundation, Vienna, Austria). Data normalization steps such as feature transformation and standardization are needed for radiomics features due to the intrinsic differences in the range, scale, and statistical distributions of these features [19]. All features were normalized with the Z-score method.

To reduce overfitting of the machine learning model, the least absolute shrinkage and selection operator (LASSO) algorithm with 10-fold cross validation was used to filter radiomics features [20]. The selected features with non-zero coefficient were then linear combined that were weighted by their respective coefficients to build a radiomics signature, here we called radiomics score (Rad-score).

### Establishment of machine learning models

In the training dataset, logistic regression algorithm was used to establish the machine learning model. Three models were built separately to predict LVSI in the training and testing datasets: the radiomics model, which only included the Rad-score parameter to predict LVSI; the protein model, which included COX-2 and TNC expression to predict LVSI; and the combined model, which included all selected parameters. In the testing dataset, models were tested and evaluated independently.

### Statistical analysis

All statistical analyses were performed with SPSS version 25.0 (IBM Corp., Armonk, NY, USA) and R version 3.5.1. The Mann-Whitney U test is used for continuous variables, and the Pearson chi-square test is used for non-continuous variables to evaluate the feature distribution of the training dataset and the testing dataset. Spearman correlation analysis of LVSI situation with immunohistochemical data and PET imaging features was performed. Mann-Whitney U test was used to distinguish differences in PET features and protein expressions between patients with early-stage cervical cancer with (LVSI group) and without (non-LVSI group) in all dataset. Multivariate logistic regression analysis was used to predict LVSI in all patients.

Receiver operating characteristic (ROC) curve was used to evaluate the performance of each machine learning model. Pairwise comparison of area under ROC curves (AUC) was performed with DeLong test. All statistical tests were performed with a two-tailed *p* < 0.05 considered statistically significant.

## Results

### Baseline characteristics of patients

The patients’ characteristics are summarized in Table 1. A total of 112 early-stage cervical cancer patients were enrolled in this study as the whole cohort and 86 cases were further distributed randomly to either the training cohort or testing cohort. Twenty-six cases were used for model validation. The training dataset included 61 patients with a median age of 50 years (range 33–74 years) and the testing dataset had 25 patients with a median age of 51 years (range 40–58 years). The external dataset included 26 patients with a median age of 52 years (range 40–74 years). Thirty patients had LVSI in the training dataset that was pathologically proven (30/61) and 12 patients (12/25) in the testing dataset. There were 15 patients with LVSI in the external dataset. There was no significant difference of all characters between the training and testing dataset (Table 1).

**Table 1:** Patient characteristics

Characteristic	Training dataset (*N* = 61)	Testing dataset (*N* = 25)	*P* value	External dataset (*N* = 26)
Age,median (range)years	50(33–74)	51(40–58)	0.970	52(40–74)
FIGO stage, No. (%)				
Ia	18(29.50)	4(16.00)		5(19.23)
Ib	26(42.60)	10(40.00)		12(46.15)
IIa	17(27.90)	11(44.00)		9(34.62)
Tumor grade, No. (%)			0.085	
Well differentiate	14(23.00)	6(24.00)		6(23.08)
Moderately differentiate	29(47.50)	17(68.00)		15(57.69)
Poorly differentiate	18(29.50)	2(8.00)		5(19.23)
Depth of cervical stromal tumor invasion, No. (%)			0.605	
≥ 1/2	43(70.50)	19(76.00)		21(80.77)
<1/2	18(29.50)	6(24.00)		5(19.23)
LVSI, No. (%)			0.921	
Yes	30(49.20)	12(48.00)		15(57.69)
No	31(50.80)	13(52.00)		11(42.31)
COX-2 expression No. (%)			0.400	
Negative or Weak-Positive	28(45.90)	11(44.00)		11(42.31)
Moderate-Positive	22(36.10)	12(48.00)		10(38.46)
Strong-Positive	11(18.00)	2(8.00)		5(19.23)
TNC expression No. (%)			0.975	
Negative or Weak-Positive	40(65.60)	17(68.00)		19 (73.08)
Moderate-Positive	16(26.20)	6(24.00)		6(23.08)
Strong-Positive	5(8.20)	2(8.00)		1(3.85)

*FIGO* International Federation of Gynecology and Obstetrics, *LVSI* lymphovascular space invasion, *COX-2* cyclooxygenase-2, *TNC* tenascin-C

### Rad-score based on PET radiomics features

**Fig. 1:**
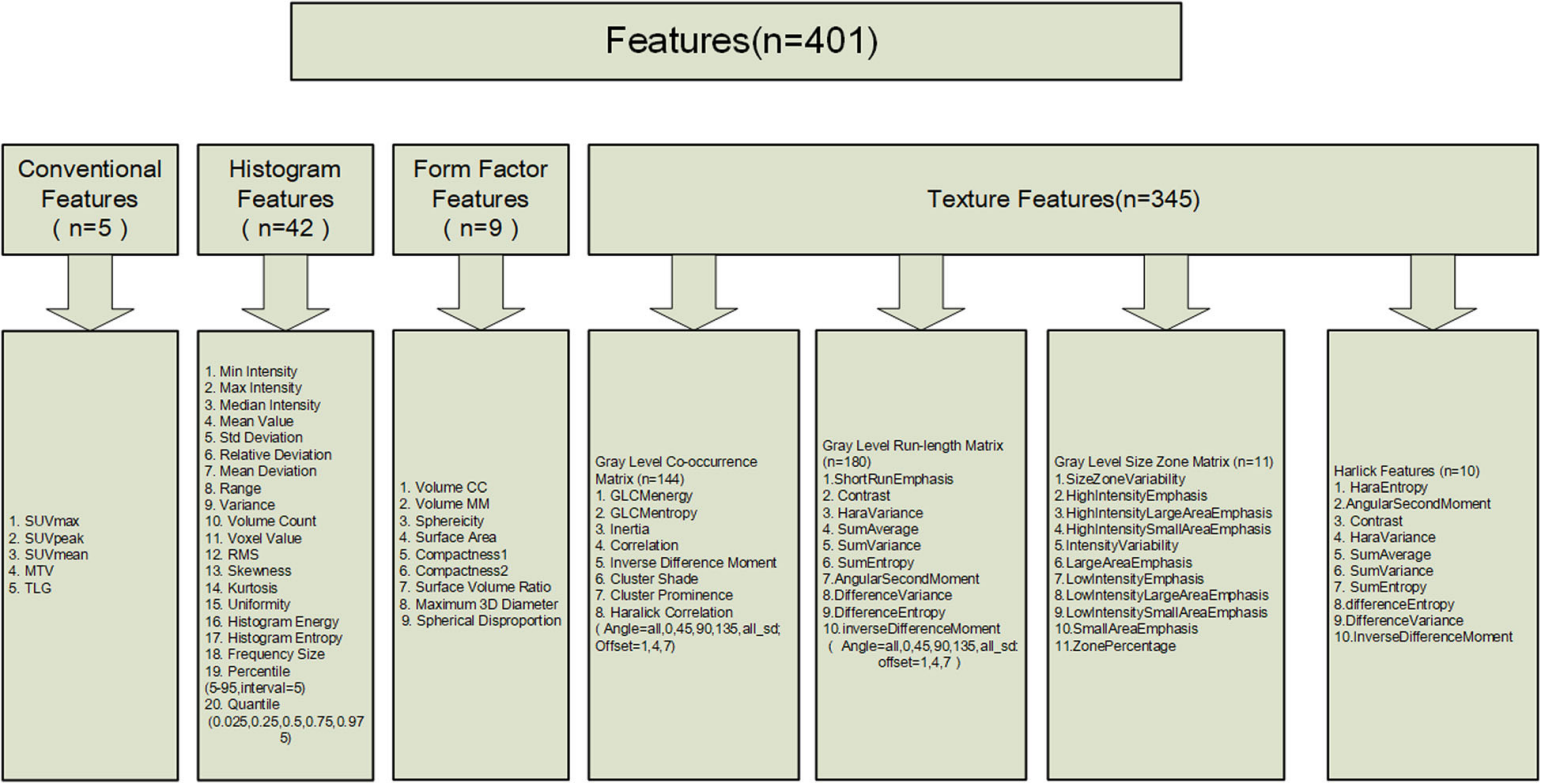
A total of 401 features were extracted from PET images, which consist of conventional features (*n* = 5), and 396 radiomics features including histogram features (*n* = 42), form factor features (*n* = 9) and texture features (*n* = 345)

**Fig. 2:**
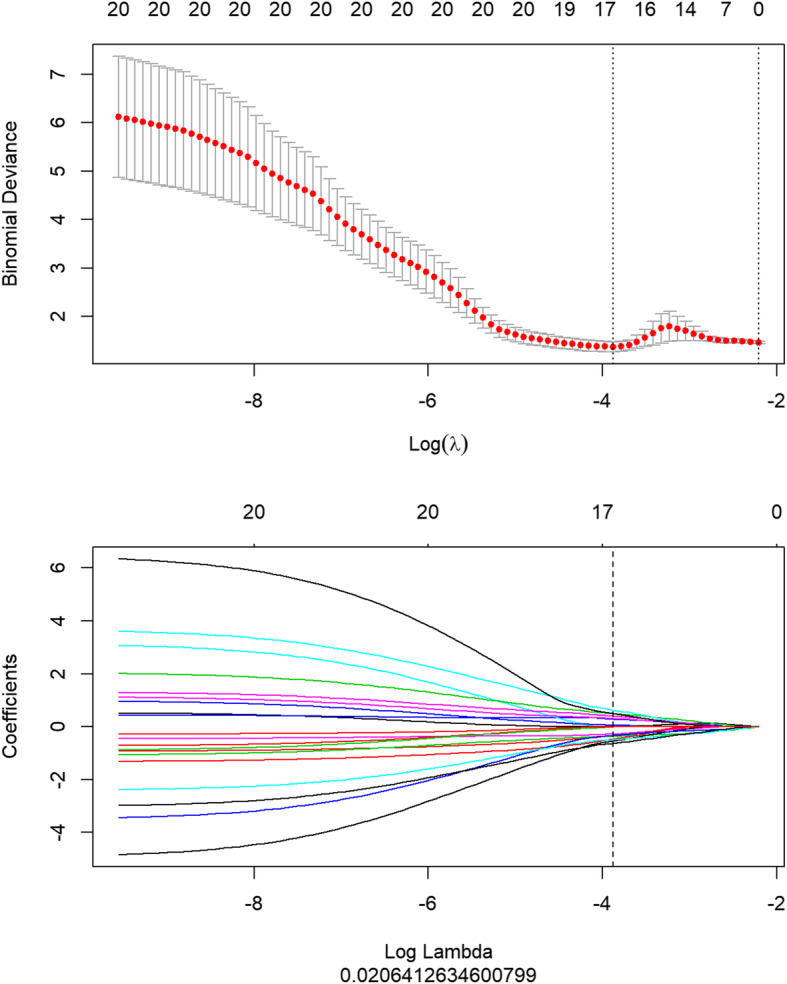
The least absolute shrinkage and selection operator was used to select informative features. When lambda was 0.02 in the 10-fold cross validation, the binomial deviance had the minimum number

**Fig. 3:**
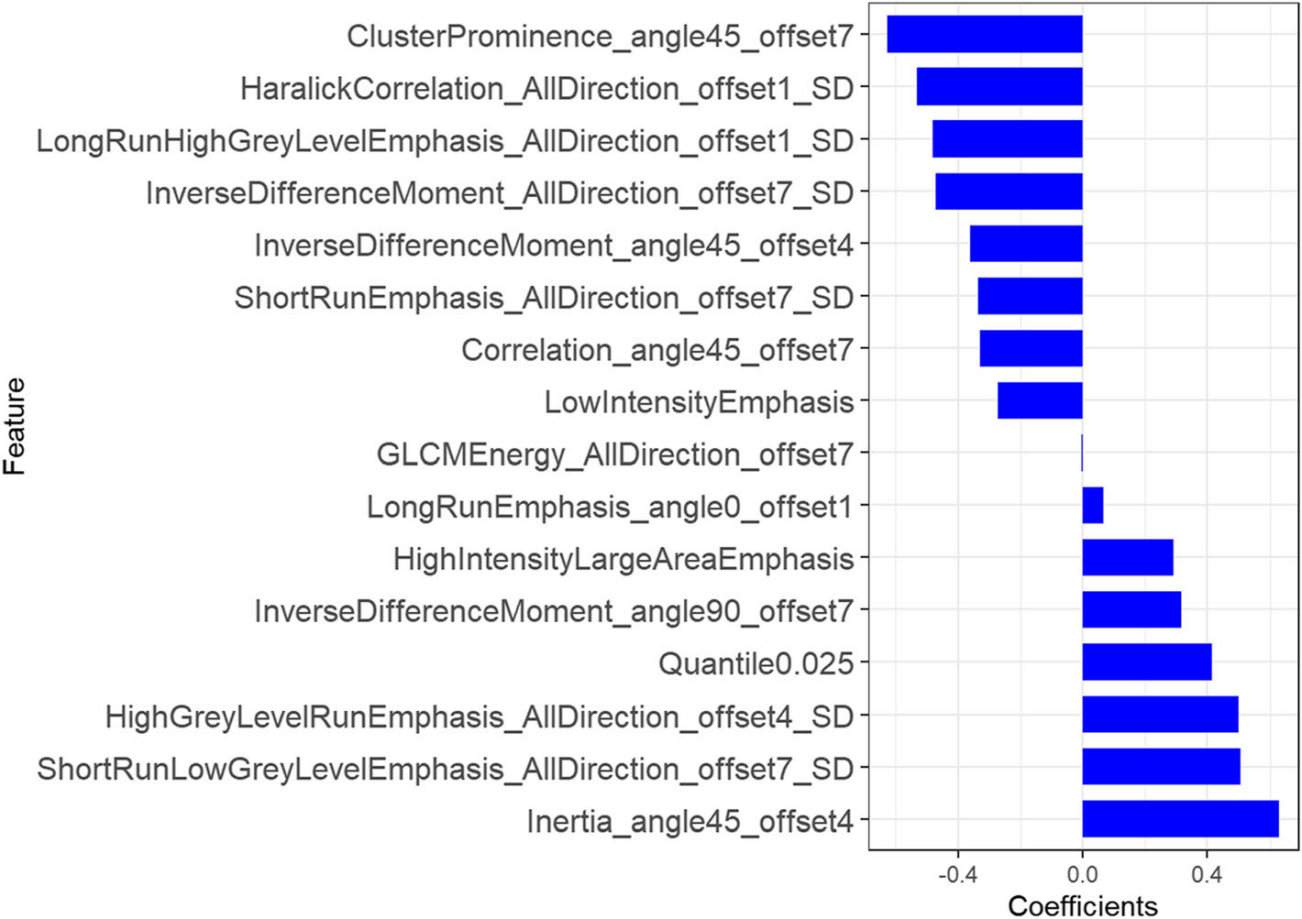
Sixteen most informative features reserved for the Rad-score calculation after least absolute shrinkage and selection operator selection. The abscissa is the size of the correlation coefficient. The ordinate represents the feature names

A total of 401 features were derived from the VOI of primary tumor on PET images that included five conventional features (SUVmax, SUVmean, SUVpeak, MTV and TLG) and 396 radiomics features (42 histogram features, 345 texture features, and 9 form factor features), as shown in Fig. 1. In the training dataset, a total of 16 most informative features with non-zero coefficient (Fig. 2) were reserved in the LASSO regression analysis when lambda chosen as 0.02(Fig. 3), which the binomial deviance was minimum in the 10-fold cross validation. So, the overfitting effect of machine learning model was the lowest. The rad-score was calculated using following formula:

$$ \mathrm{Rad}-\mathrm{score}\ \left(\mathrm{PET}\right)=-0.328\ast \mathrm{Correlation}\_\mathrm{angle}45\_\mathrm{offset}7-0.361\ast \mathrm{InverseDifferenceMoment}\_\mathrm{angle}45\_\mathrm{offset}4+0.291\ast \mathrm{HighIntensityLargeAreaEmphasis}-0.273\ast \mathrm{LowIntensityEmphasis}-0.531\ast \mathrm{HaralickCorrelation}\_\mathrm{AllDirection}\_\mathrm{offset}1\_\mathrm{SD}-0.47\ast \mathrm{InverseDifferenceMoment}\_\mathrm{AllDirection}\_\mathrm{offset}7\_\mathrm{SD}+0.499\ast \mathrm{HighGreyLevelRunEmphasis}\_\mathrm{AllDirection}\_\mathrm{offset}4\_\mathrm{SD}-0.335\ast \mathrm{ShortRunEmphasis}\_\mathrm{AllDirection}\_\mathrm{offset}7\_\mathrm{SD}-0.482\ast \mathrm{LongRunHighGreyLevelEmphasis}\_\mathrm{AllDirection}\_\mathrm{offset}1\_\mathrm{SD}+0.415\ast \mathrm{Quantile}0.025-0.626\ast \mathrm{ClusterProminence}\_\mathrm{angle}45\_\mathrm{offset}7+0.065\ast \mathrm{LongRunEmphasis}\_\mathrm{angle}0\_\mathrm{offset}1+0.63\ast \mathrm{Inertia}\_\mathrm{angle}45\_\mathrm{offset}4+0.316\ast \mathrm{InverseDifferenceMoment}\_\mathrm{angle}90\_\mathrm{offset}7+0.507\ast \mathrm{ShortRunLowGreyLevelEmphasis}\_\mathrm{AllDirection}\_\mathrm{offset}7\_\mathrm{SD}+-0.004\ast \mathrm{GLCMEnergy}\_\mathrm{AllDirection}\_\mathrm{offset}7-0.075 $$

### Correlation of LVSI with PET features and molecular protein expressions

**Fig. 4:**
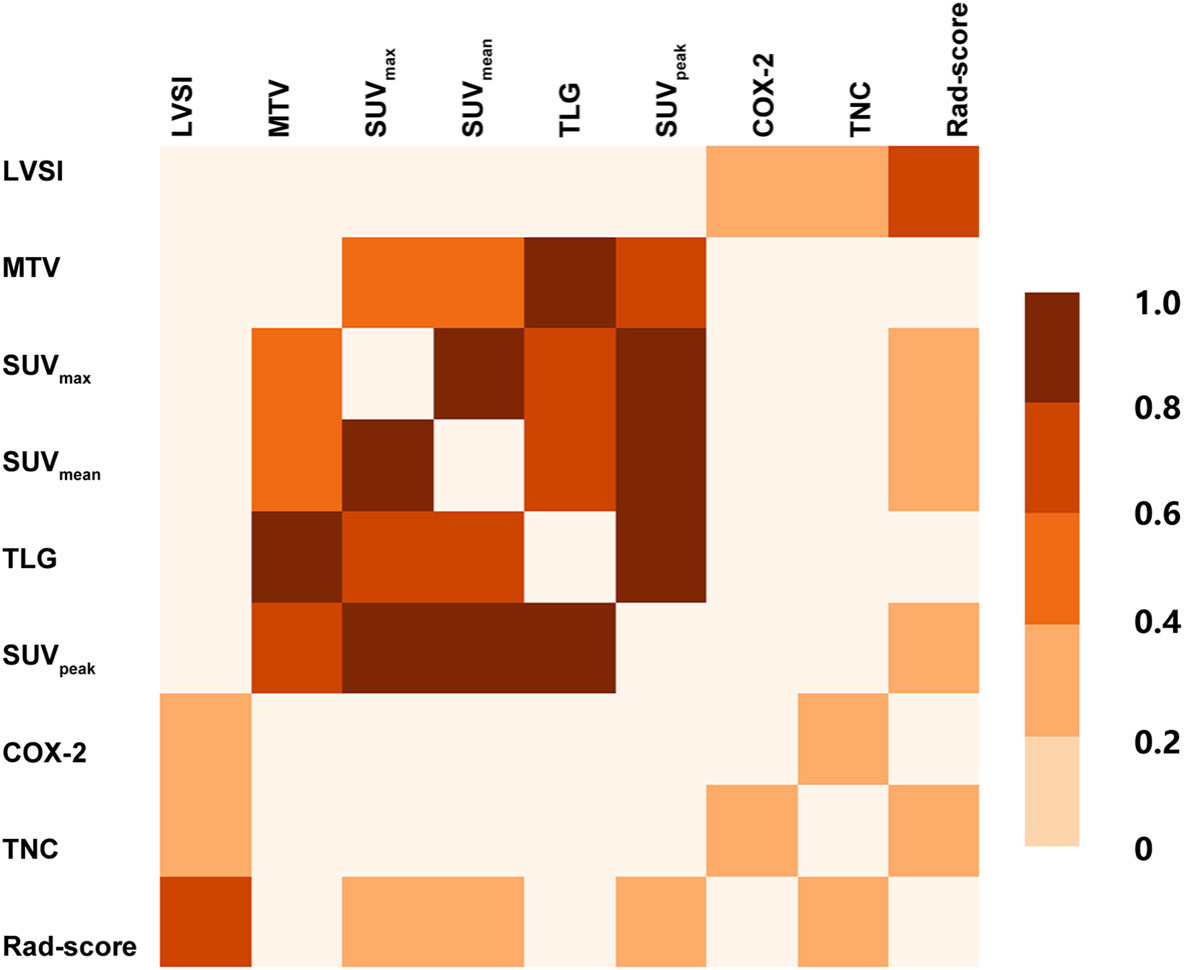
Heatmap of the correlation of LVSI, Rad-score, conventional parameters, TNC, and COX-2. The color scale on the right represents the interval of statistically significant regression coefficients. White squares indicate no statistical significance (*p* > 0.05). LVSI, lymphovascular space invasion; MTV, metabolic tumor volume; SUV_max_, maximum of the standard uptake value; SUV_mean_, mean standard uptake value; TLG, total lesion glycolysis; SUV_peak_, peak standard uptake value; COX-2, cyclooxygenase-2; TNC, tenascin-C; Rad-score, radiomics score

The Spearman correlation analysis revealed a significant correlation between LVSI and the Rad-score (*r* = 0.631, *p* < 0.001) (Fig. 4). LVSI was also correlated to COX-2 and TNC expression (*r* = 0.276, *p* = 0.01, and *r* = 0.333, *p* = 0.002, respectively). The other PET conventional parameters did not have significant correlation with LVSI or protein expressions. TNC was correlated to both the Rad-score (*r* = 0.244, *p* = 0.024) and COX-2 (*r* = 0.227, *p* = 0.036).

### Univariate analyses of the PET features and molecular protein expression with LVSI

**Fig. 5:**
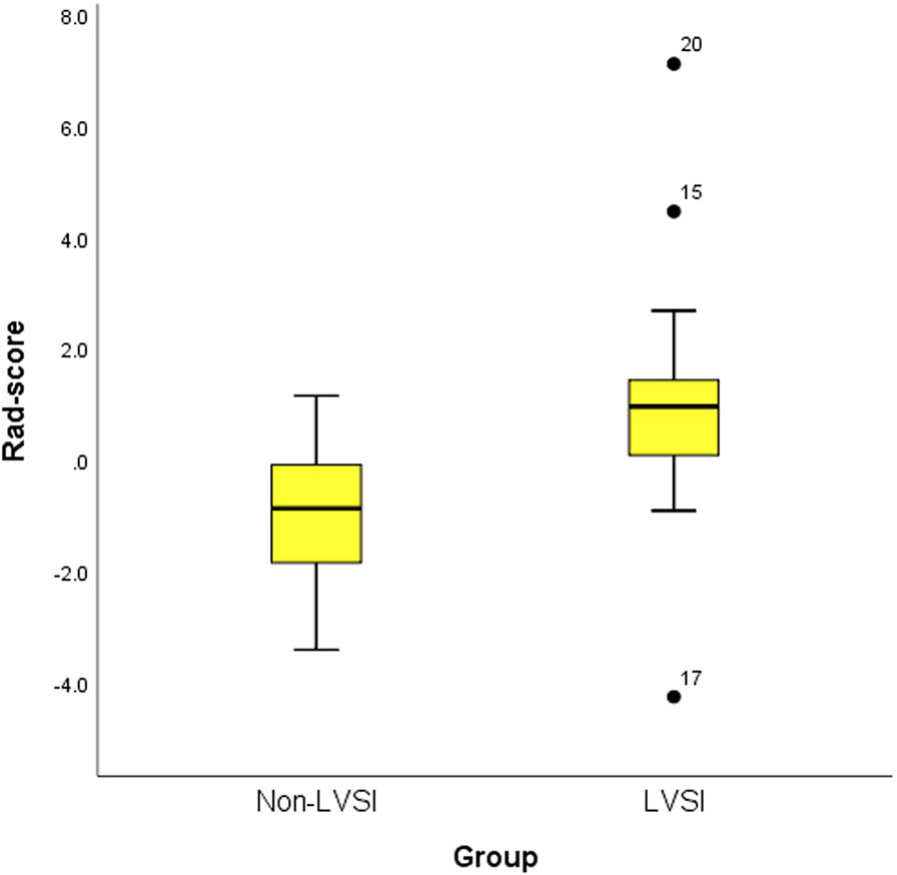
The Rad-score of patients with LVSI was significantly higher than those without LVSI. Rad-score (PET), radiomics score derived from positron emission tomography imaging

The results of the Mann-Whitney U test are summarized in Table 2. The Rad-score, COX-2, and TNC significantly differed between the LVSI and non-LVSI groups (*p* < 0.005). The LVSI group had a higher Rad-score than the non-LVSI group (*p* < 0.001, Mann-Whitney U test) (Fig. 5).

**Table 2:** Differences between the LVSI and non-LVSI groups in all dataset

Variable	COX-2	TNC	SUV_**max**_	TLG	SUV_**peak**_	Rad-score (PET)
Mann-Whitney U	594.000	517.000	877.500	896.000	894.500	251.000
Wilcoxon W	1584.000	1507.000	1867.500	1886.00	1884.500	1241.000
Z	−3.096	−3.886	−0.402	−0.242	− 0.255	− 5.814
p	0.002	< 0.001	0.688	0.809	0.799	< 0.001

Mann-Whitney U test was used to distinguish differences between the LVSI and non-LVSI groups

*COX-2* cyclooxygenase-2, *TNC* tenascin-C, *SUV*_max_ maximum of the standard uptake value, *TLG* total lesion glycolysis, *SUV*_peak_ peak standard uptake value, *Rad-score (PET)* radiomics score derived from positron emission tomography imaging

### Prediction of LVSI in the multivariate logistic regression analysis

In the multivariate logistic regression analysis, only the Rad-score and TNC were associated with LVSI in all patients with early-stage cervical cancer (Table 3).

**Table 3:** Multivariate logistic regression analysis

Variable	Coefficient	SE	Wald	p
TNC	1.36497	0.45012	9.1958	0.0024
Rad-score (PET)	1.20806	0.30471	15.7186	0.0001
Constant	−0.88808	0.38653	5.2789	0.0216

*SE* standard error, *TNC* tenascin-C, *Rad-score (PET)* radiomics score derived from positron emission tomography imaging

### Evaluation of the machine learning models

**Fig. 6:**
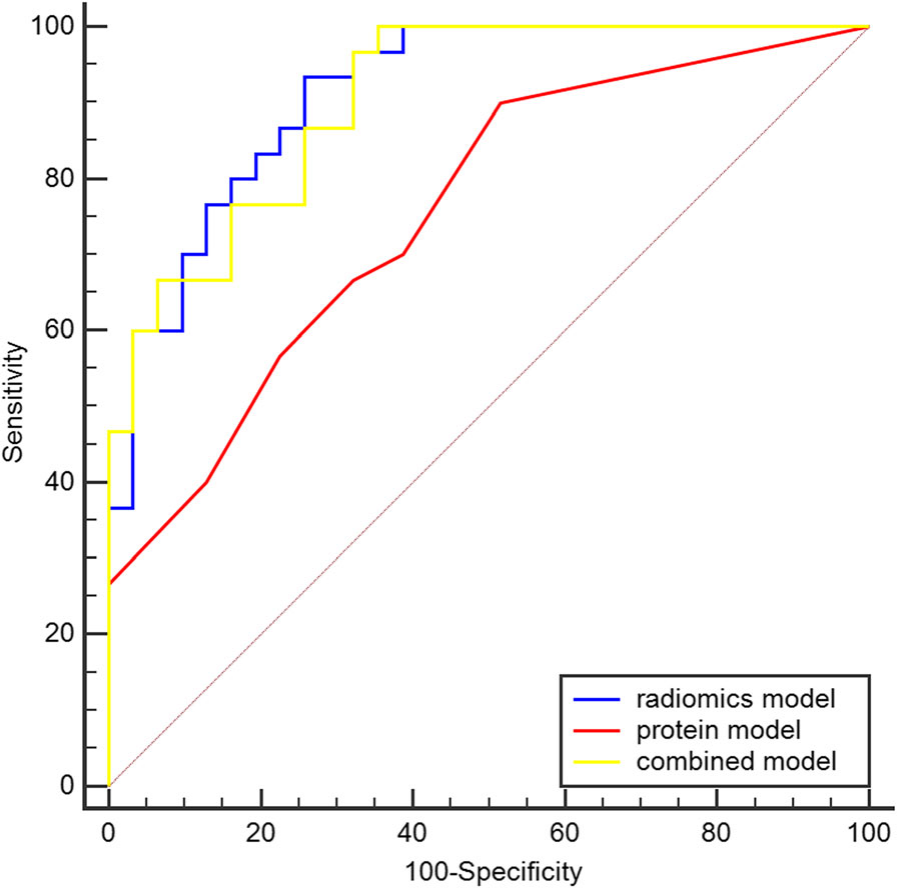
Receiver operating characteristics curves of the radiomics, proteins, and combined models in the training dataset. Blue curve, radiomics model; red curve, protein model; yellow curve, combined model

The ROC curve was used to evaluate the efficiency of the machine learning models. In the training dataset, three models all performed well in predicting LVSI (Table 4). The radiomics model had the best predictive performance among the models (AUC = 0.914; 95% confidence interval, CI, 0.814–0.970; *p* < 0.001) in the training dataset. The combined model had high sensitivity in predicting LVSI in the training dataset (sensitivity = 1.0; specificity = 0.64; *p* < 0.001) (Fig. 6).

**Table 4:** Performance of each model in the training and testing datasets

Model	Training dataset				Testing dataset				External dataset			
	AUC (95% CI)	***p***	Sen	Spe	AUC (95% CI)	***p***	Sen	Spe	AUC (95% CI)	***p***	Sen	Spe
**Radiomics model**	**0.914 (0.814–0.970)**	**< 0.001**	**0.93**	**0.74**	**0.756 (0.545–0.904)**	**0.014**	**0.58**	**0.92**	**0.806 (0.606–0.933)**	**< 0.001**	**0.80**	**0.82**
**Protein model**	**0.756 (0.630–0.857)**	**< 0.001**	**0.90**	**0.48**	**0.631 (0.417–0.814)**	**0.238**	**0.50**	**0.77**	**0.503 (0.302–0.703)**	**0.979**	**0.87**	**0.27**
**Combined model**	**0.905 (0.803–0.965)**	**< 0.001**	**1.00**	**0.64**	**0.801 (0.594–0.932)**	**0.003**	**0.67**	**1.00**	**0.782 (0.577–0.918)**	**0.002**	**0.53**	**1.00**

*AUC* area under the curve, *CI* confidence interval, *Sen* sensitivity, *Spe* specificity

**Fig. 7:**
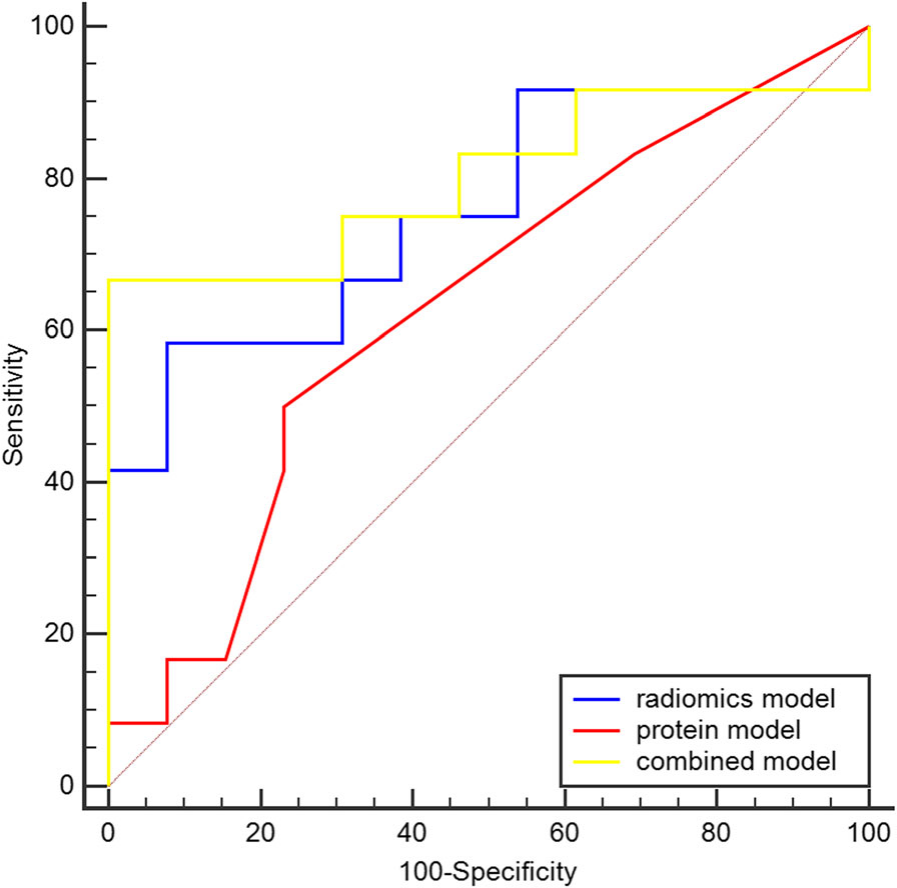
Receiver operating characteristics curves of the radiomics, protein, and combined models in the testing dataset. Blue curve, radiomics model; red curve, protein model; yellow curve, combined model

However, in the testing dataset, the combination of radiomics, COX-2, and TNC for predicting LVSI had better efficiency than the other models (AUCs: 0.801 vs. 0.756 and 0.801 vs. 0.631, respectively; sensitivity = 0.67; specificity = 1.00). The performance of the protein model was not statistically significant (AUC = 0.631, *p* = 0.238) (Fig. 7).

**Fig. 8:**
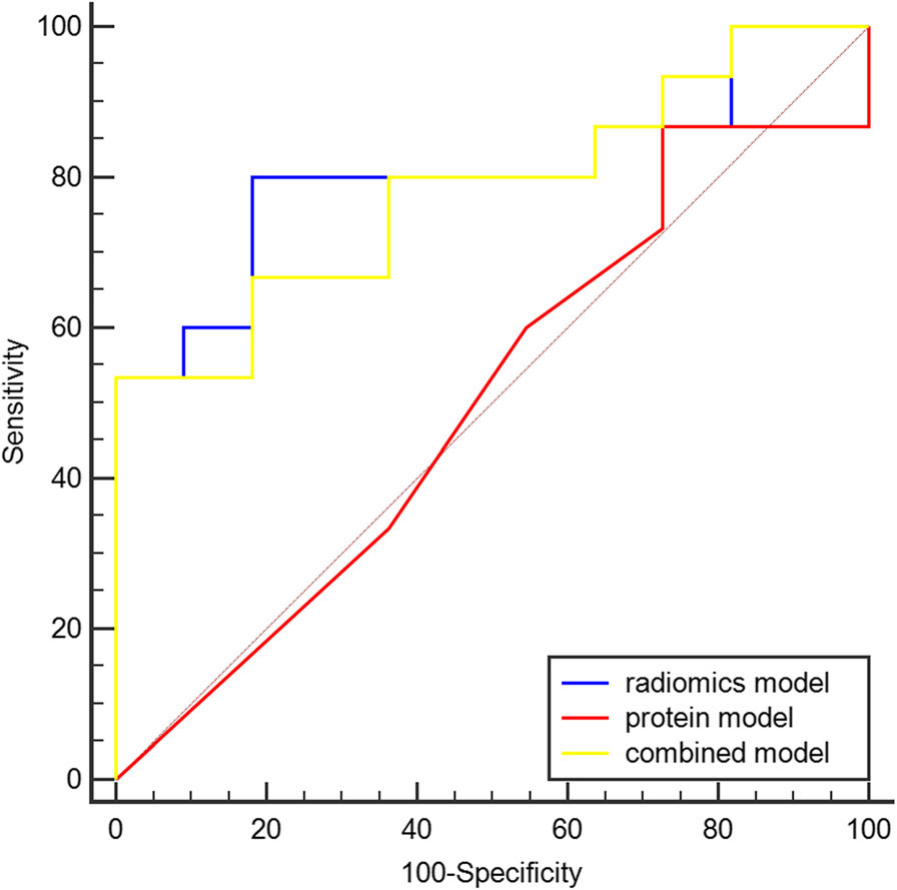
Receiver operating characteristics curves of the radiomics, protein, and combined models in the external dataset. Blue curve, radiomics model; red curve, protein model; yellow curve, combined model. The area of ROC curve of radiomics model was better than the others’ area

The results of evaluating for all models in the external dataset were shown in the Table 4. In the external dataset, the radiomics model had the best performance for predicting LVSI (AUC = 0.806, *p* < 0.001) (Fig. 8). However, the specificity of combined model was best of all models in the external dataset.

### Pairwise comparison of the ROC curves of the models

In the training dataset, the AUC of the ROC of the combined model was better than that of the protein model. Furthermore, the radiomics model provided better prediction performance than the protein model (Table 5). However, in the testing and external dataset, the result of the DeLong test showed that the AUC of ROC curves of the three models were not significantly different(*p* > 0.05) (Tables 6 and 7).

**Table 5:** DeLong test of ROC curve of models in the training dataset

Pairwise comparison of ROC curves	Difference between areas	Z	p
Radiomics and protein models	0.158	2.196	0.028^*^
Radiomics and combined models	0.008	0.619	0.536
Protein and combined models	0.149	2.317	0.021^*^

*ROC* receiver operating characteristics

*Statistically significant, *p* < 0.05

**Table 6:** DeLong test of ROC curve of models in the testing dataset

Pairwise comparison of ROC curves	Difference between areas	Z	*p*
Radiomics and protein models	0.125	0.794	0.427
Radiomics and combined models	0.045	1.265	0.206
Protein and combined models	0.170	1.192	0.233

*ROC* receiver operating characteristics

**Table 7:** DeLong test of ROC curve of models in the external dataset

Pairwise comparison of ROC curves	Difference between areas	Z	*p*
Radiomics and protein models	0.303	1.943	0.052
Radiomics and combined models	0.024	0.918	0.358
Protein and combined models	0.279	1.640	0.101

### Reproducibility of tumor segmentation

**Fig. 9:**
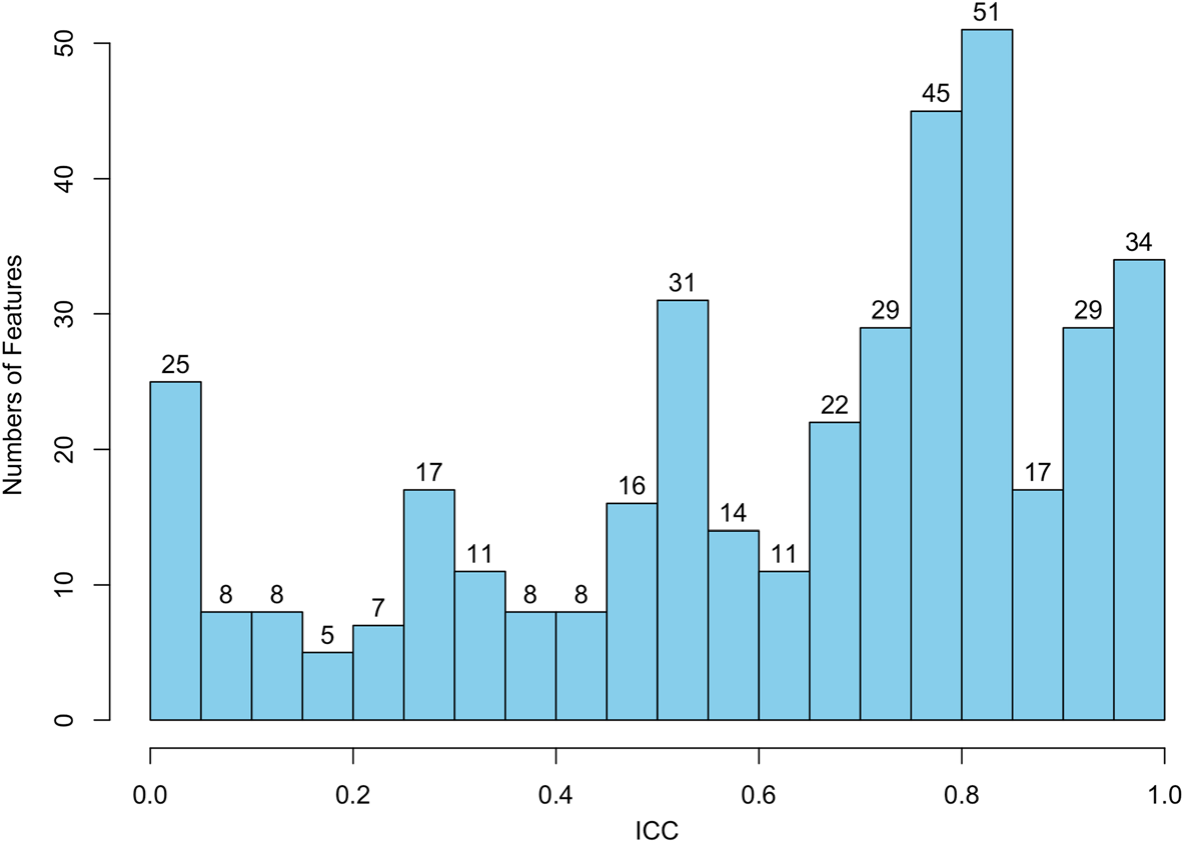
The histogram showed the ICC value of radiomics features. The ICC value of 176 radiomics features was higher than 0.75 (*p* < 0.05)

The ICC was used to evaluate the reproducibility of tumor segmentation on PET images. The result of ICC analysis was shown in the histogram (Fig. 9). The histogram showed that the ICC value of 176 radiomics features was higher than 0.75(*p* < 0.05).

## Discussion

According to the 2018 FIGO criteria [15], surgery is the primary treatment for patients with early-stage squamous cervical carcinoma. LVSI has an important influence on surgery and patient prognosis according to the FIGO criteria [15]. Although most patients have excellent prognosis, approximately 30% patients might have recurrence and decreased survival rate [21]. Many studies have proven that LVSI is closely associated with prognosis and is an independent risk factor [2–4, 6, 7]. The expression of some proteins, including COX-2, TNC, and others, are related to LVSI, tumor microenvironment, and inflammation [11, 12, 22]. Hence, accurate and early assessment of LVSI is important in prognosis assessment and treatment decision making in order to ensure that patients can obtain the maximum treatment benefit.

Our study aimed to establish a machine learning model that combines radiomics derived from PET images with molecular proteins that are associated with the pathology of cervical cancer in order to predict LVSI in patients with early-stage cervical cancer. The results indicate that the Rad-score was closely correlated with LVSI, and there were statistically significant differences in COX-2, TNC, and the Rad-score between the LVSI and non-LVSI groups. Moreover, we determined that the model based on the Rad-score could predict LVSI. When the Rad-score and molecular protein expression were combined, the AUC of the model improved a bit in the testing dataset, but the DeLong test showed no statically significant difference between the two models in the testing dataset.

Malignant tumors exhibit intratumoral biological heterogeneity and lead to changes in the texture parameters of the corresponding primary tumor on PET images. A previous study determined that heterogenic FDG uptake within a tumor correlated with intratumoral histopathological appearance [23]. Several researches proved that the texture information of tumors reflected the tumors’ heterogeneity [24, 25]. The selected radiomics features of our study were also texture parameters of PET image. Mu et al. [26] found that inverse difference moment and correlation showed statistically significant differences between the early (stages I and II) and advanced stages (stages III and IV) of cervical cancer. Similarly, inverse difference moment and correlation were selected to calculate the Rad-score for predicting LVSI in our study. Recently, Li et al. [18] study showed that the PET textures of primary tumor could predict lymphatic metastasis in early-stage cervical carcinoma (AUC = 0.757 in the validation dataset; 95% CI, 0.545–0.904; *p* < 0.05). Other research have also shown that radiomics of primary tumor based on PET images could reflect tumor malignancy and were associated with nodal metastases and molecular subtypes of solid tumor [27, 28]. The Rad-score [29], which is calculated by the linear combination of selected features (including histogram and texture parameters) weighted by their respective coefficients selected as informative features, is usually used for radiomics analysis. In the present study, the Mann-Whitney U test showed that the Rad-score and TNC had significant differences between the LVSI and non-LVSI in all datasets, and the LVSI group had a higher Rad-score than the non-LVSI group in Fig. 5.

A previous research found that the molecular expression of some proteins was correlated with LVSI. Normal cervical tissues have weak expression of TNC and COX-2. Previously, Pilch et al. [13] determined that, in invasive cervical carcinoma, TNC expression was markedly increased. Other studies have proven that TNC has a significant role in tumor growth, migration, metastasis, angiogenesis, and stromal inflammation [14, 30, 31]. The study of Liu et al. [11] found that COX-2 expression was associated with lymphangiogenesis and lymph node metastasis in cervical cancer. Similarly, Hoellen et al. [12] proved that COX-2 expression was significantly associated with LVSI (*p* = 0.017). Similarly, our study also found that the differences in COX-2 and TNC between the LVSI and non-LVSI groups were statically significant. In the multivariate logistic regression, TNC expression was associated with LVSI. Furthermore, COX-2 had a slight correlation with TNC (Fig. 4). We hypothesized that the result may be caused by the inflammatory microenvironment of the tumor. Liu et al. [11] demonstrated that COX-2 may promote cancer progression and metastasis by enhancing the expression of vascular endothelial growth factor C and other mechanisms. The research showed that TNC could also facilitate the formation of cancer stroma, including desmoplasia and angiogenesis, and enhanced inflammation in the cancer stroma may augment macrophage recruitment and secretion of tumor-promoting and inflammatory cytokines by macrophages and fibroblasts [14]. Thus, TNC and COX-2 were selected to predict LVSI in cervical cancer.

Although some protein expression and radiomics were associated with LVSI in cervical carcinoma, the correlation of molecular proteins and radiomics has been rarely explored and confirmed. In our study, the Rad-score was correlated with TNC, according to the Spearman correlation analysis (Fig. 4). Thus, we assumed that the PET imaging textures of primary tumors changed through TNC and other proteins. The textures of primary tumor reflect the heterogeneity of tumor, and they could be used to predict LVSI in cervical carcinoma. Another research also hoped to utilize the expression of TNC on PET imaging by devising a new PET tracer [32]. However, our result initially showed that radiomics derived from PET imaging provided a new possibility for non-invasive visualization of TNC expression. Song et al. [33] also found that the image signal changes on magnetic resonance imaging (MRI) were consistent with TNC expression, and cervical cancer tissues with node metastasis had the highest TNC expression.

Three machine learning models were established with logistic regression algorithm in the training dataset and evaluated in the testing dataset. All three models performed well in the training dataset (Table 4), but the radiomics model had the highest AUC in the training and external dataset (Figs. 6 and 8). However, in the testing dataset, the AUC value of the combined model was higher than that of the other models (Table 4). The reason for the results was that our dataset was slightly smaller. Thus, we used all datasets to perform the multivariate logistic regression analysis (Table 3). The results also showed that the Rad-score and TNC were associated with LVSI in all datasets. Two different methods (statistics and machine learning) both confirmed that the combination of radiomics and TNC could predict LVSI in early-stage cervical cancer. In the external dataset, the combined model for predicting LVSI was also credible and the specificity of combined model was 100%. But the AUC of radiomics model was the best among three models in the external dataset. The DeLong test also indicated that the AUC of the ROC of the combined model was better than that of the protein model in the training dataset (Table 5). Previously, a few researchers also wanted to predict or distinguish LVSI through radiology for cervical cancer. Yang et al. [34] determined found that the minimum apparent diffusion coefficient and the minimum apparent diffusion coefficient ratio were significantly lower in LVI-positive invasive cervical cancer than in LVI-negative invasive cervical cancer (0.772 ± 0.062 vs. 0.917 ± 0.052, *p* < 0.001, and 0.712 ± 0.078 × 10^− 3^ vs. 0.867 ± 0.099 × 10^− 3^ mm^2^/s, *p* < 0.001, respectively). Gross tumor volume [35] on MRI was also identified to be a possible independent risk factor for predicting LVSI (AUC = 0.700, *p* < 0.05). Recently, the use of radiomics based on magnetic resonance for predicting LVSI has been studied. According to Hua et al. [36], the model based on multiparametric MRI showed the best prediction results, with an AUC of 0.842 (95% CI, 0.772–0.913; sensitivity = 0.773; specificity = 0.776) in the training cohort and 0.775 (95% CI, 0.637–0.912; sensitivity = 0.739; specificity = 0.667) in the validation cohort. Similarly, Li et al. [37] also found that the radiomics nomogram derived from MRI showed favorable discrimination between LVSI and non-LVSI groups, with an AUC of 0.754 (95% CI, 0.6326–0.8745) in the training cohort and 0.727 (95% CI, 0.5449–0.9097) in the validation cohort. We initially used the combination of PET radiomics with protein molecule to predict LVSI, which showed that the radiomics and combined models based on ^18^F-FDG PET imaging showed better results than those of previous studies.

For patients with early-stage cervical cancer, when LVSI is evident, pelvic lymphadenectomy should be considered, along with modified radical hysterectomy according to the FIGO criteria [15]. However, pelvic lymphadenectomy may cause a series of postoperative complications including lower limb lymphedema, bladder dysfunction and others [38–40]. Combined model and radiomics model of our search could predict LVSI with PET image and histological data before surgical treatment. When the machine learning model predicts that LVSI is negative, patients are likely to avoid being subjected to pelvic lymphadenectomy. Individualized medical treatment with radiomics can improve the postoperative life quality of patients.

However, this study has some limitations. First, the size of the dataset was inadequate; thus, we need a larger number of dataset to test our models as well as multicenter imaging data to evaluate reproducibility. Second, we only analyzed the association of the expression of TNC and COX-2 with LVSI. In the future, we hope to perform more protein analyses and explore the correlation of DNA with LVSI and the function of radiogenomics in order to predict LVSI. Finally, the PET image resolution was low, thus limiting the precision of the segment of tumor VOI as well as the extraction of the radiomics features.

## Conclusion

The results of this study indicated that LVSI in patients with early-stage cervical cancer can be predicted by radiomics derived from PET imaging. The machine learning model that combines PET-based radiomics with COX-2 and TNC provides a new tool for detecting LVSI. The combined model improved prediction accuracy. In addition, the Rad-score derived from PET image textures is associated with TNC expression. In the future, the models of this study needs to be tested by a larger number of patients in multiple centers so that it can be used in clinical treatment.

## Data Availability

The datasets used and analyzed of the current study are available from the corresponding author on reasonable request.

## Abbreviations

LVSI: Lymphovascular space invasion
COX-2: Cyclooxygenase-2
TNC: Tenascin-C
FIGO: International Federation of Gynecology and Obstetrics
MRI: Magnetic resonance imaging
AUC: Area under ROC curves
ROC: Receiver operating characteristic
Rad-score: Radiomics score
LASSO: Least absolute shrinkage and selection operator
